# Corrigendum

**DOI:** 10.3892/ol.2013.1309

**Published:** 2013-04-17

**Increased cyclin-dependent kinase 6 expression in bladder cancer**

GANG WANG, LINGYAN ZHENG, ZHIJIAN YU, GUODONG LIAO, LIQIN LU, RUJUN XU, ZHONGSHENG ZHAO and GUANGDI CHEN

Oncology Letters 4: 43–46, 2012; DOI: 10.3892/ol.2012.695

After the publication of the article, the authors noted that they had made an error regarding certain data in their manuscript.

On page 44, ‘The monoclonal mouse anti-Cdk6 antibody (1:50 dilution; Millipore, Billerica, MA, USA) was used for primary antibody incubation at 4˚C overnight’ should be replaced with ‘The monoclonal mouse anti-Cdk6 antibody (1:50 dilution; Abcam, Cambridge, MA, USA) was used for primary antibody incubation at 4˚C overnight’.

